# Investigation on Hysteretic Behavior of Embedded PVC Pipe Confined Reinforced High Strength Concrete Columns

**DOI:** 10.3390/ma13030737

**Published:** 2020-02-06

**Authors:** Zongping Chen, Yuhan Liang, Xuebing Zhao, Ji Zhou

**Affiliations:** 1 College of Civil Engineering and Architecture, Guangxi University, Nanning 530004, China; LIANGYUHAN2017@163.com (Y.L.); 1803110140@st.gxu.edu.cn (X.Z.); 17687657937@163.com (J.Z.); 2 Key Laboratory of Disaster Prevention and Structure Safety of Chinese Ministry of Education, Guangxi University, Nanning 530004, China

**Keywords:** PVC, confined reinforced high-strength concrete, reinforced high-strength concrete, columns, seismic performance, shear bearing capacity

## Abstract

To study the seismic performance of embedded polyvinyl chloride (PVC) pipe confined reinforced high-strength concrete (PVC-RHC) columns, five specimens are designed for cyclic loading test, which include three PVC-RHC column specimens, an embedded circle steel tube confined reinforced high-strength concrete (CST-RHC) column specimen, and a reinforced high-strength concrete (RHC) column specimen. The failure mechanism and morphology are revealed by experiments. The influences of PVC pipe diameter, axial compression ratio, and concrete strength on seismic performance indexes are analyzed. The research results indicate thhe following: all specimens displayed shear baroclinic failure. Compared with RHC specimens, the hysteretic curves of the PVC-RHC specimen and CST-RHC specimen were fuller; furthermore, their energy dissipation capacity, deformation, and ductility were more beneficial. With the increase of the diameter–length ratio and axial pressure, the energy dissipation capacity and deformation capacity of PVC-RHC specimens decreased. The shear bearing capacity of the PVC-RHC specimen calculated with “concrete structure design code” (GB 50010-2010) was smaller than the test results by 25%, showing an excessive safety margin. Thus, according to the failure mechanism of the PVC-RHC specimen, a new calculation formula of shear bearing capacity is deduced, which is in good agreement with the experimental results.

## 1. Introduction

Owing to the advantages of high strength, little deformation, and good durability, reinforced high-strength concrete (RHC) is widely used in long-span, heavy-duty, and high-rise buildings [1,2,3]. However, with the increase of concrete strength, the frangibility increases and plastic deformation ability decreases. Usually, the volume–stirrup ratio is improved in order to increase the deformation capacity of RHC, which also leads to the construction difficulties, the increased use of steel and high cost of projects [4,5]. Therefore, it is necessary to seek more effective and economical ways. On the basis of the theory of a concrete-filled steel tube, a new type of confined high-strength concrete column embedded with polyvinyl chloride pipe (PVC-RHC) is proposed, which is intended to reduce the density of the stirrup and ensure good ductility by the confined effect of the PVC pipe to core concrete [6,7].

Large relevant universities and research institutes have also carried out some tests on PVC pipe-constrained concrete. Through the axial compression test of a PVC plastic pipe confined concrete column, C.E.Kurt found that the PVC plastic pipe improved the mechanical properties of core concrete by providing a constraining force [8]. It is proposed that the PVC plastic pipe confined concrete column can be applied to engineering. H.Toutanji [9,10] systematically studied the mechanical properties, durability, and design methods of grooved PVC-FRP (Fiber Reinforced Polymer) pipe concrete short columns. Other researchers [11,12,13,14,15,16] found that PVC pipe confined concrete mostly appears plastically damaged, which improves the bearing capacity, seismic deformation, and durability of specimens with less cost, energy saving, and environmental protection. At the same time, the confinement mechanism of concrete-filled steel tubular columns should be discussed. Patel VI [17] performed a parametric analysis of fiber-based models to examine the effects of local buckling, bending axis, aspect ratio, and material strength on uniaxial compression RCFST (Round-ended concrete-filled steel tubular) short beam-columns’ performance.

However, the relevant research of the PVC-RHC has not been reported in the literature at present. In order to reveal its failure mechanism and seismic performance, the low-cyclic loading test of the PVC-RHC is carried out. It is expected to provide new technical support for the further promotion and application of high-strength concrete.

## 2. Experimental Program

### 2.1. Specimen Design and Preparation

Five short-column specimens were designed for the low-cyclic loading test, including three PVC-RHC specimens (PVC-RHC-1−PVC-RHC-3), a circle steel tube (CST)-RHC specimen, and an RHC specimen. The PVC pipe diameter (Φ110 × 3, Φ75 × 2.3), axial compression ratio (0.25, 0.45), and concrete strength (75.01 MPa, 79.44 MPa) were considered as the main parameters. The parameters of the specimens are shown in Table 1.

**Figure 1:**
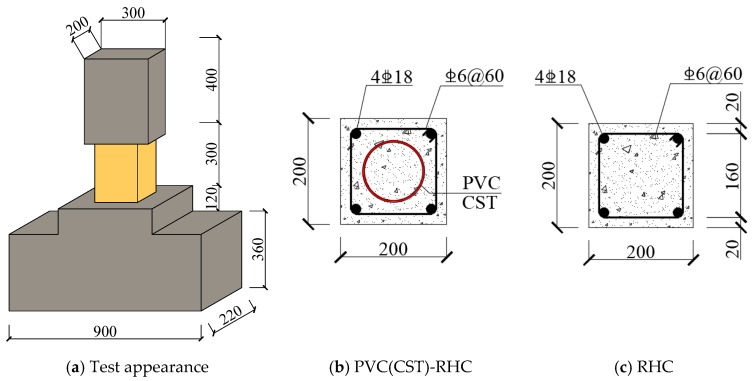
Cross-section dimension and reinforced distribution. PVC, polyvinyl chloride; CST, circle steel tube; RHC, reinforced high-strength concrete.

**Figure 2:**
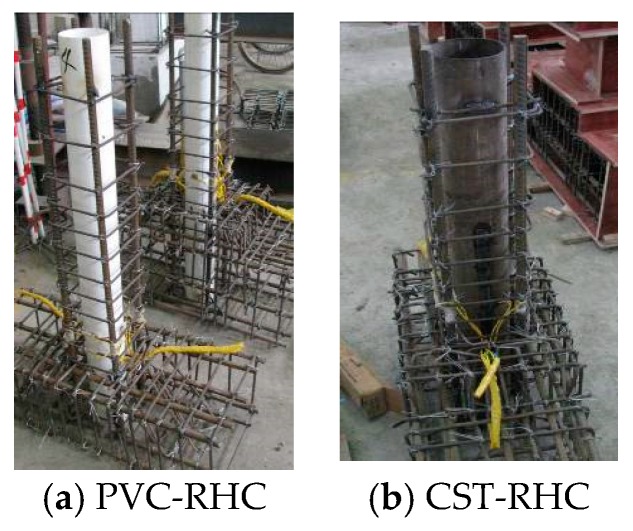
Internal skeleton of specimens.

All specimens have the same external dimensions. The cross-section of the test section was 200 × 200 mm with the height of 300 mm. The size of the loading end was 200 × 300 × 400 mm, which was embedded with four M24 high-strength bolts for connection with the actuator. The size of the fixed end was 900 × 220 × 360 mm, where the horizontal longitudinal bar was 14 mm HRB400 (hot-rolled ribbed bars, and the standard value of the yield strength of the steel bar is 400 MPa), and the stirrup was 8 mm HRB335 with spacing of 50 mm. The longitudinal rebar and stirrup were HRB400 and HRB335, respectively, with the ratio of 2.543% and 1.273%, respectively. The cross-section and reinforcement situation of specimens are shown in Figure 1. The internal skeletons of the specimens are shown in Figure 2.

### 2.2. Material Properties

The main constituent materials are described as follows:

- Ordinary portland cement of 42.5 MPa.
- Medium coarse river sand.
- Crushed rock aggregate, which had a maximum size of 20 mm and a minimum size of 5 mm.

**Figure 3:**
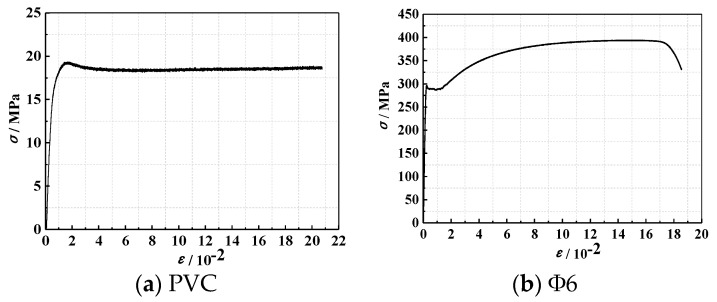
Material stress–strain curves.

In the test, high-strength concrete was stirred by mixer in the laboratory on the spot. AF-C polycarboxylic acid superplasticizer (Huangteng building materials chemical co.LTD, Nanning, China) was adopted in the tests. The column members and the corresponding concrete test block strength tests are performed at the same time. The average compressive strength of the high-strength concrete cube and the concrete mix proportion are shown in Table 2. *f*_cu_ represents the cube compressive strength of concrete. Table 3 lists the physical properties of steel and the PVC pipe, in which *f*_y_ and *f*_u_ represent the yield strength and ultimate strength, respectively. *E*_s_ is the elastic modulus. The physical properties determined on steel and the PVC pipe were obtained using the MTS (Mechanical testing and simulation) electro-hydraulic servo testing machine (MTS industrial systems (China) co. LTD, Shanghai, China), as shown in Figure 3.

### 2.3. Test Setup and Loading Process

**Figure 4:**
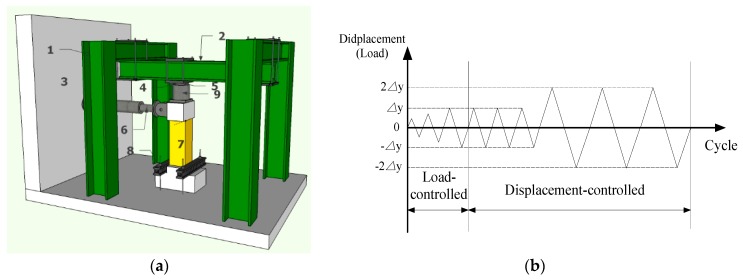
(**a**) Test set up (1. Reaction steel frame; 2. Reaction steel beam; 3. Reaction wall; 4. Spherical hinge; 5. High strength rolling shafts; 6. Electro-hydraulic servo actuator; 7. Specimen; 8. Fixed steel beam; 9. Hydraulic jack.) and (**b**) loading process.

A cantilever-type pseudo-static loading device was used in the test. In the loading process, the vertical load was applied and kept constant through the hydraulic jack, and then the horizontal repeated load was applied by the electro-hydraulic servo actuator of 500 kN. The loading process includes the load-controlled phase and displacement-controlled phase, as shown in Figure 4. The specimens were loaded according to the load control with 10 kN as the load increment before yielding, and the load was cycled once per grade. When the specimen first yielded, the loading process entered the displacement-controlled phase. The displacement of each stage was circulated three times with the yield displacement as increment. The test finishes until the lateral loads’ resistance deteriorates to 85% of the maximum measured lateral loads.

**Figure 5:**
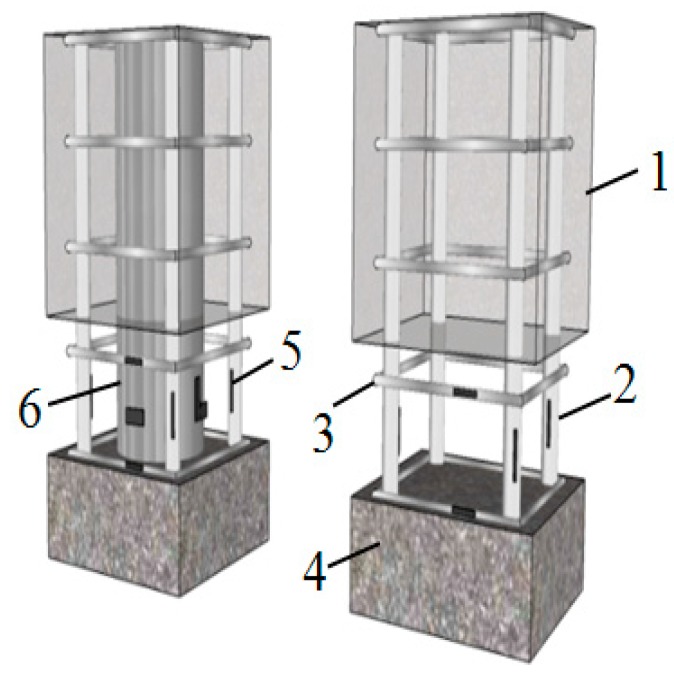
Gauge points arrangement (1. Concrete; 2. Longitudinal Reinforcement; 3. Stirrup form; 4. Fixed end; 5. Gauge points; 6. PVC (CST).).

The horizontal load and displacement of the cylinder top are collected automatically by the electro-hydraulic servo actuator control system. The strain of concrete, steel bar, and PVC pipe is collected by Strain Collection DH3820 (Donghua testing technology co. LTD, Jiangjiang, China). The arrangement of strain gauges is shown in Figure 5. In order to record the crack development of the specimen, a grid was drawn on the surface of the specimen. The crack development at each stage of loading was recorded by hand drawing and on-site photography.

## 3. Experiment Results and Analysis

### 3.1. Failure Modes

**Figure 6:**
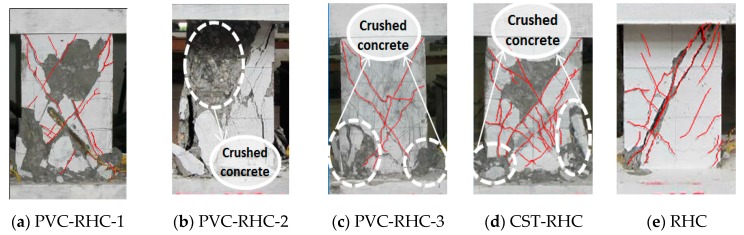
Failure pattern of specimens.

The cracks and failure modes are shown in Figure 6. All specimens were characterized as inclined shearing failure mode. At the beginning of loading, the specimen was in the elastic stage. With the increase of load, most of the specimens began to show slight oblique cracks within 200 mm away from the bottom of the column, among which the horizontal bending cracks first appeared in the specimens of PVC-RHC-2 with high axial pressure ratio and CST-RHC with internal steel tube. Continuing loading, it can be seen that the horizontal bending cracks appeared on the side of the column. At the same time, the existing horizontal bending cracks developed slowly under the constraint of the core column, and a large number of oblique cracks gradually appeared in the abdomen. Another inclined crack extended to both ends and formed a cross inclined crack, which divided the surface of the specimen into several prismatic concrete blocks. There are slight vertical cracks in the bottom corner of the compression side column. In the high axial pressure specimens, a small amount of concrete also had the phenomenon of spalling, owing to press damage. When the load increased to the peak, the specimen surface formed a few obvious main cross inclined cracks, and the intersection of most of the stirrups achieved the yield strength. Owing to the friction between the cracks’ occlusion effect and restriction in the core of the confined concrete column, however, the bearing capacity was still stable, until the concrete block similar to prismatic was crushed, the large area of stirrups was bare, and the specimens’ bearing capacity dropped and they were rapidly destroyed.

Furthermore, this test component mainly consists of three parts—the external reinforced concrete, the pipe, and the concrete core pillar inside the pipe. In destructing, the external reinforced concrete part is cracked first. Then, the surface concrete falls off and the reinforcement is exposed. As the apparent change of the inner steel pipe (PVC pipe) and the core column cannot be observed directly during the test, it can be seen through the analysis of the test points and the numerical simulation verification analysis of the seismic performance test of the high-strength concrete column restrained with the PVC pipe by the Abaqus method. After the external reinforced concrete part is out of work, with the progress of the load, for the PVC pipe core column, when the PVC pipe reaches the limit, the internal high-strength concrete has not reached the strength limit. For the steel pipe core column, the steel pipe reaches first. After yielding, the internal concrete was crushed and the test specimen reached its limit state.

It can be seen from the failure morphology of the specimens that, except for the reinforced high-strength concrete column specimens restrained by ordinary stirrup, the final failure morphology of all the specimens presented diagonal cross oblique cracks. Because of the existence of the internal core of confined concrete columns with steel tubes, after cracking, the reinforced high-strength concrete column specimens confined with pipes formed many thin bending cracks and oblique cracks, which can prevent or reduce the appearance of main cracks and avoid the situation of forming main cracks quickly in the reinforced high-strength concrete columns confined with ordinary stirrup constraints, and ductility and energy dissipation capacity are obviously improved.

### 3.2. Hysteresis Curve

**Figure 7:**
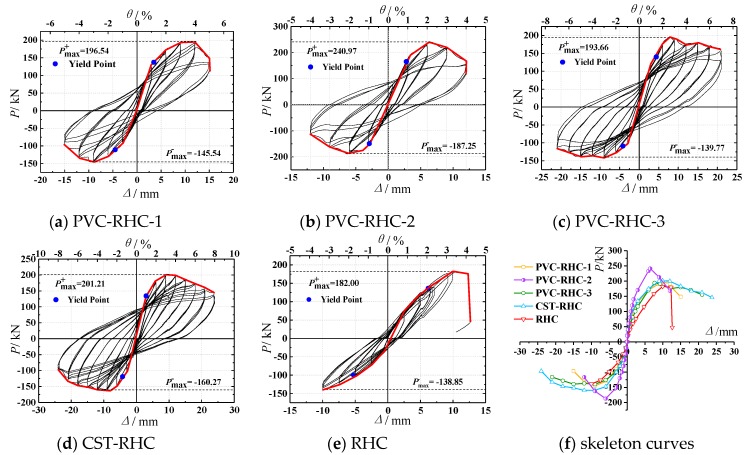
Hysteretic loops and skeleton curves of the specimen.

Figure 7 shows the load–displacement hysteresis curve measured of the specimens. As can be seen from the figure, the hysteretic curves of the embedded PVC pipe represented the following. From the beginning of loading to the elastic stage before concrete cracking, the area of hysteresis loop was narrow and slender, and the load basically changed linearly with the displacement. After entering the elastic–plastic stage, along with the increase of the crack in the specimen, the curvature of the loading and unloading curves of the specimen gradually decreased, the stiffness gradually degraded, and the displacement hysteresis after unloading was gradually obvious. In the displacement control stage, with the increase of horizontal displacement, the bearing capacity continued to increase. Before the displacement reached the peak load, the three cyclic curves of the same displacement amplitude basically coincided with each other without obvious intensity attenuation. After reaching the peak load of the specimen, the enveloped area of the hysteretic ring further increased, the strength and stiffness obviously degraded, and the plastic deformation increased. Under the same displacement amplitude, each cycle had different degrees of strength attenuation compared with the previous cycle, and the hysteretic ring shape was approximately fusiform.

Compared with the reinforced high-strength concrete specimens, the hysteretic curves of the embedded PVC pipe and steel tube confined reinforced high-strength concrete specimens were fuller and the deformation capacity was stronger, among which the embedded steel tube specimen appeared the most significant, indicating that the measures of the embedded PVC pipe can enhance the energy dissipation capacity and deformation capacity of the reinforced high-strength concrete short columns.

Compared with PVC-RHC-1, PVC-RHC-3 with a smaller diameter of the PVC pipe had a fuller hysteresis curve, stronger energy dissipation capacity, and slower intensity decay in the later stage. It can be seen that the seismic energy dissipation and deformation capacity of the specimen are significantly affected by the PVC pipe diameter. It is also very important to consider a reasonable area ratio of the PVC pipe concrete core column in the design stage. Under the condition of similar strength concrete, the area enveloped by hysteretic curve of PVC-RHC-2 of a higher axial compression ratio was larger than that of the specimen PVC-RHC-1, but the bearing capacity was less stable and the deformation ability was weakened in the later period; with the increase of shear span ratio, the hysteresis curve of the specimen was longer and narrower, the energy dissipation capacity was weakened, and the ultimate deformation was small, indicating that the seismic deformation ability of specimen decreased with the increase of the axial compression ratio and shear span ratio.

### 3.3. Skeleton Curve

After connecting the peak points of each level of loading in the same direction, the skeleton curves of each specimen were obtained as shown in Figure 7f. As can be seen from the figure, before the crack appeared, the slope of the skeleton curve of each specimen did not change significantly with the increase of load. After cracks appeared on the surface of the specimen, it entered the stage of elastic–plastic deformation. With the failure of concrete and the yield of steel, the slope of the skeleton curve gradually decreased with the increase of the load value until it reached the peak load. After the skeleton curve entered the descending section, most of the specimens showed good ductility. It can be seen from the figure that the skeleton curves of each specimen were not completely symmetric with respect to the origin, which was mainly because of the residual deformation and accumulated damage of the specimen after positive loading, which needed to be offset during reverse loading, so in order to reach the expected deformation, the load required value needed to be smaller.

As can be seen from the figure, the ratio of peak load to yield load of all specimens was between 1.16 and 1.58, with a high safety reserve. For the reinforced high strength concrete column specimens restrained by ordinary stirrup, after reaching the peak load, the bearing capacity stability was weaker, the descending section was steep, and the deformation ability was bad. After embedding pipes, it can be seen that the peak load of the reinforced high-strength concrete column specimens PVC-RHC-1 and CST-RHC restrained with the embedded PVC pipe and steel tube increased by 8.0% and 10.6%, respectively, compared with that of the RHC specimen. Moreover, the descending section of the skeleton curve was more gentle, the limit displacement increased, and the ductility performance was improved. In particular, the late deformation performance of the specimens embedded with the steel pipe was improved more significantly.

Comparing the skeleton curve of the specimen PVC-RHC-1 with that of the specimen PVC-RHC-3, it can be seen that, with the increase of the PVC pipe diameter, the initial stiffness of the specimen also increased, but the peak load did not increase much, about 1.5%. After the peak value, it dropped steeply, the strength attenuation was accelerated, the stiffness degraded obviously, and the deformation ability became worse in the later stage. This may be because the wall of the PVC pipe was smooth and the bond strength of concrete was small. When the PVC pipe diameter increased, the thickness of the concrete cover accordingly became thin, which likely produced the bond slip effect between the PVC pipe and concrete. The two parts of concrete inside and outside the PVC pipes could not work well, causing the earlier specimen cracking, but with a larger area of large diameter constraint of the core concrete, the initial stiffness was larger. As the horizontal load increased to the peak, the lateral deformation of the core concrete increased sharply inside the tubes, owing to the extrusion of the PVC pipe wall, the expending effect of the peripheral expansion concrete protective layer was obvious, leading to thinner prismatic concrete block convex drum crushing to fall off, which caused the bearing capacity to decrease and fail. In the experiment, it can also be seen that, in the PVC-RHC-1 specimen, the concrete dropped largely.

Under the condition of approximation with concrete strength, when the axial compression ratio *n* increased from 0.25 to 0.45, the specimen peak load increased by 22.6%, but the stiffness degradation and intensity attenuation was accelerated, the limit deformation was reduced, and ductility became worse. This is because, when improving the axial compression ratio appropriately, it made it slow and limited the internal micro cohere emergence and development of cracks, improved the aggregate interlocking, strengthened the bolt pin effect of longitudinal reinforcement, and raised the shear bearing capacity of the components. However, after the fracture development was inhibited, the internal savings of energy dissipation cannot be dissipated. The *P*-*Δ* effect was more significant because of the large deformation in the late load. This caused the principal stress and principal compressive strain of concrete to increase sharply, weakening the plastic deformation capacity of the concrete and, at the same time, the internal energy was suddenly released, leading to the rapid destruction of the specimen and worse ductility.

## 4. Seismic Performance Index and Influence Factor Analysis

### 4.1. Ultimate Bearing Capacity

**Figure 8:**
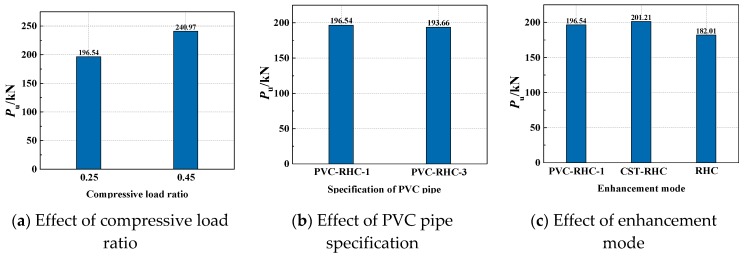
Parametric analysis of bearing capacity.

The measured bearing capacity and displacement of the specimens are shown in Table 4. The influence of each parameter on the ultimate bearing capacity of the specimens is shown in Figure 8. It can be seen from the figure that the bearing capacity of the specimens increases with the increase of the axial compression ratio in the test interval. This is because the high-strength concrete column with the embedded PVC pipe is passively restrained to the core concrete, thus causing the concrete to be affected. The greater the axial pressure, the more significant the Poisson effect and the stronger the restraining effect.

The cracking load and yield load of the soil column specimens were about 54.9% and 71.5% of the peak load, respectively; those of the PVC-RHC specimens were about 51% and 71.7%, respectively; and those of the CST-RHC specimens were 59.6% and 66.7%, respectively. It can be seen that the cracking load was slightly reduced by using the PVC pipe in the ordinary stirrup confined high-strength concrete column. The steel tube can restrain or slow down the cracking of concrete, but the yield load reduced.

### 4.2. Ductility Coefficient

The ductility coefficient and displacement angle between the layers of each specimen are also given in Table 5. The ductility coefficient is calculated as follows:

(1) $$\mu =\frac{\Delta_{u}}{\Delta_{y}}$$

where *Δ*_u_ is for ultimate displacement, with a decline period of 0.85 times the peak load of the displacement. *Δ*_y_ is the yield displacement, based on the general bending moment method.

**Figure 9:**
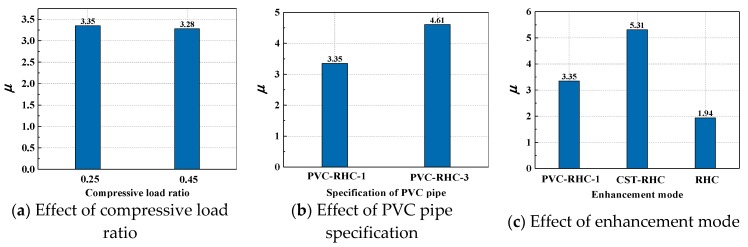
Parametric analysis of ductility.

It can be seen from the table that the displacement ductility coefficient of the reinforced high-strength column restrained by the ordinary stirrup is 1.94, while the displacement ductility coefficient of the reinforced high-strength specimen restrained by the embedded PVC pipe is within the range of 3.28−4.61, most of which is greater than 3, that is, the ductility of reinforced high strength column restrained by the embedded PVC pipe is better than that restrained by the ordinary stirrup. Figure 9 shows the influence of each design parameter on the ductility performance of high-strength concrete columns constrained by the internal PVC pipe. It can be seen from the figure that, compared with the specimens with the embedded PVC pipe, the specimens with the embedded steel pipe have an enhanced deformation capacity and superior ductility performance when the bearing capacity is not significantly reduced. When other conditions are the same, with the increase of the PVC pipe diameter and axial pressure ratio, the ductility of the specimen gradually becomes worse.

When cracking, the elastic interlayer displacement angle and limit interlayer displacement angle of all specimens were 1/173−1/76 and 1/48−1/15, respectively, both of which exceeded the limit of the interlayer displacement angle in the code, indicating that reinforced high-strength concrete columns have strong lateral and collapse resistance. Compared with the specimen RHC, the reduction rate of the elastic inter-layer displacement angle mean value of PVC-RHC-1 and PVC-RHC-3 of specimens was slower, and the increase in the amplitude of the limit elastic–plastic inter-layer displacement angle mean value was larger, indicating that the PVC pipe equipped with an appropriate pipe diameter could enhance the deformation ability of components in the later stage, improving its collapse resistance.

### 4.3. Energy Dissipation

**Figure 10:**
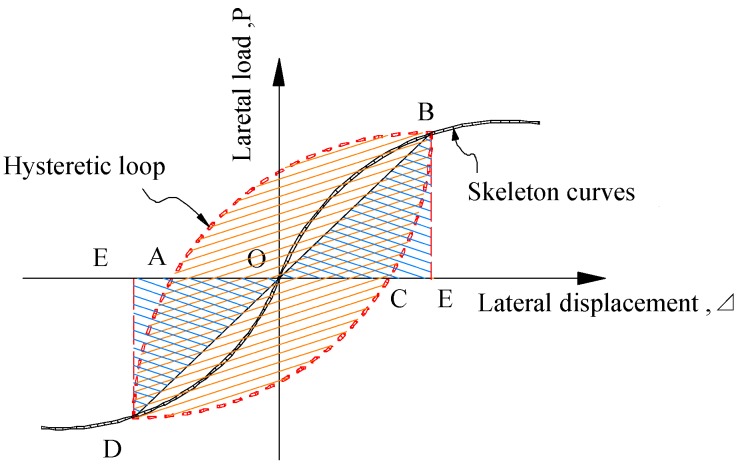
Calculation diagram of the equivalent damping ratio.

**Figure 11:**
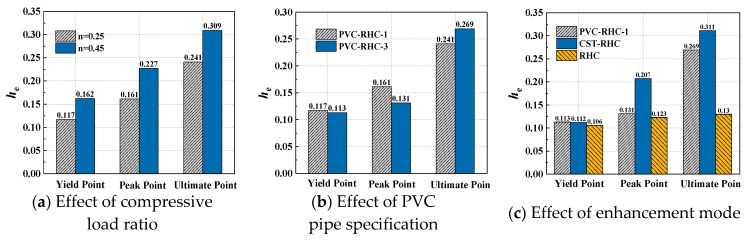
Parametric analysis of the equivalent damping coefficient.

The energy dissipation capacity of a structure or component is an important factor for judging its seismic performance. The equivalent damping ratio *h*_e_ is usually used, as shown in Figure 10. The equivalent damping ratio is calculated using Equation (2). The influence of various design parameters on the energy dissipation capacity of a specimen is shown in Figure 11.

(2) $$h_{e}=S_{\left(ABC+CDA\right)}/(2\pi \cdot S_{\left(OBE+ODF\right)})$$

As can be seen from the figures, the viscous damping coefficient *h_e_* of each specimen tends to increase with the increase of displacement, that is, the energy dissipation capacity is good in the later stage. The equivalent viscous damping coefficient *h_e_* (between 0.241 and 0.309) is higher than that of the ordinary stirrup (*h*_e_ = 0.130), which indicates that the measure of the reinforced high-strength concrete embedded PVC pipe column can significantly improve the energy dissipation capacity of the specimen. The equivalent viscous damping coefficient of the specimen CST-RHC is the highest, which indicates that the energy-dissipation capacity of high-strength concrete with the steel tube is stronger. For the specimens that are both equipped with PVC pipes, the later stage energy consumption capacity of the smaller-diameter PVC-RHC-3 is stronger than that of the larger-diameter specimen PVC-RHC-1. In addition, under the condition of approximately the same strength concrete, the energy consumption of the specimen increases with the increasing axial pressure ratio. This is because the axial pressure is small, and the lateral strain of concrete is also small, so steel tubes in the constraints of the effect are not apparent. In the scope of the experiment, with the increase of axial pressure, the constraint effect of core concrete is enhanced, so the energy dissipation performance of the specimens is enhanced with the increase of the ratio of axial compression.

### 4.4. Strength Degeneration

**Figure 12:**
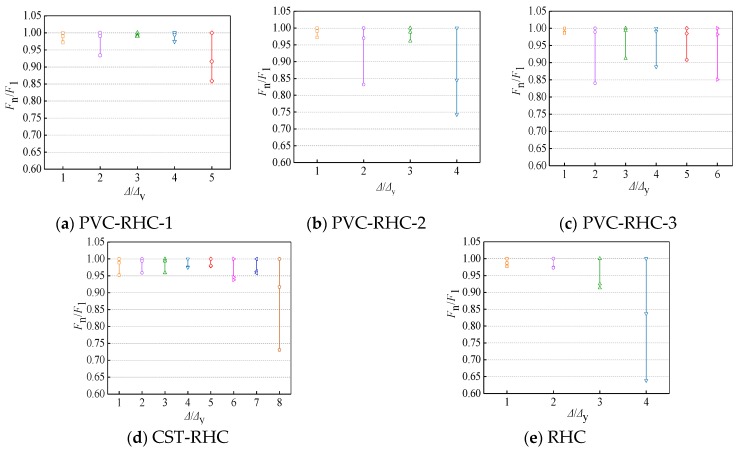
Strength degradation curves.

The ratio *F*_n_/*F*_1_ between the maximum horizontal load in the cycle and the maximum horizontal load in the first cycle at the same displacement amplitude is used to express the strength attenuation of the specimen under this cycle. The component intensity attenuation degree of intensity attenuation coefficient is *λ*, and the attenuation coefficient of the measured part of the specimen strength with the displacement ratio (*Δ*/*Δ*_y_) changes in the relationship is shown in Figure 12.

It can be seen from the figure that the strength attenuation of all specimens is accelerated with the increase of loading displacement and loading cycle times, indicating that the specimens’ ability to continue to resist the seismic action decreases after being subjected to a certain seismic action. The main reason is that, with the increase of loading displacement and cycle times, the bearing capacity of concrete decreases slowly in the early stage of loading owing to the gradual cracking of concrete; in the later stage, the concrete spalling and compression falls off and the steel damage accumulates seriously. As a result, the effective bearing area of components decreases sharply, leading to the rapid decline of bearing capacity. Along with the increase of the displacement, despite the concrete crushing loss of large area, in the late loading, the intensity attenuation of the reinforced high-strength concrete embedded with the PVC pipe and steel tube columns is still relatively slower than reinforced high-strength concrete. Among them, the strength attenuation of embedded steel tube specimens is more stable, showing that the distribution of the PVC tube and steel tube can effectively confined the core concrete to improve its performance, causing the bearing capacity to slow down and improving the stability of the intensity attenuation. With the increase of the axial compression ratio, the stability of strength attenuation decreases. PVC-RHC-2 had a lower number of loading cycles and a lower limit displacement than PVC-RHC-2 before failure, and the range of bearing capacity attenuation under the action of each cycle increased.

### 4.5. Stiffness Dissipation

**Figure 13:**
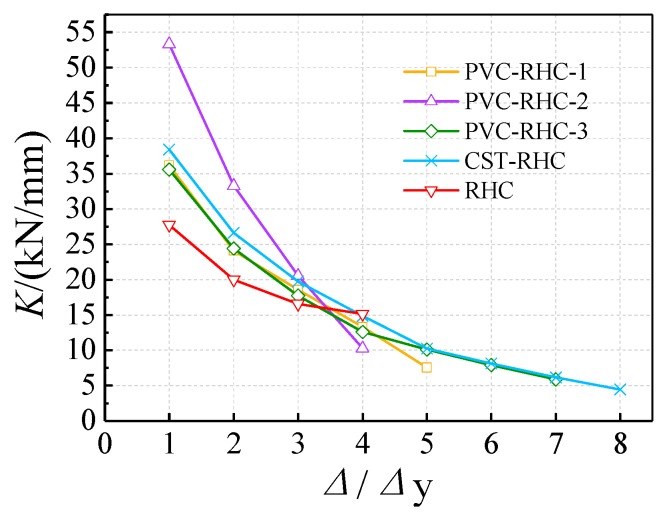
Stiffness degradation curves.

The secant stiffness *K* represents the stiffness changes of specimens. Figure 13 shows the specimen stiffness degradation with the change of the displacement ratio (*Δ*/*Δ*_y_) curve.

As can be seen from the figure, with the increase of the displacement amplitude, the stiffness of each specimen presented a trend of first fast and then slow degradation. This is mainly because, in the early stage of loading, a large number of oblique and bending cracks appeared in the root of the specimen, which made the early stiffness degradation faster. As the load continues to increase, the damage degree of concrete increases and gradually drops out of work. At this time, cracks are fully developed and almost no new cracks appeared. The plastic deformation of steel increased, which further reduced the stiffness of the members. The initial stiffness of each specimen varied greatly. The specimens with a large axial compression ratio had the highest initial stiffness, which was about 1.92 times that of the specimens with a large axial compression ratio. When *Δ*/*Δ*_y_ equals 3, all the stiffness of the specimens were greater than 35% of the initial stiffness. When loading to the failure point, the average residual stiffness of all specimens except ordinary reinforced high-strength concrete specimens is greater than 21%, indicating that the lateral stiffness of reinforced high-strength concrete confined with the PVC pipe and steel tube column had a strong stability, and the influence is most significantly affected by the axial compression ratio.

## 5. Calculation and Formula Derivation of Ultimate Shear Bearing Capacity

### 5.1. The Shear Ultimate Bearing Capacity Calculated by the Standard Formula

At present, there are no reports on the calculation methods for the ultimate bearing capacity of high-strength concrete columns embedded with PVC pipes. Owing to the complex shearing mechanism of reinforced concrete specimens and considering the low tensile strength of PVC pipes, in order to facilitate engineering applications, the structure in accordance with China’s current “Code for Design of Concrete Structures” (GB 50010-2010) [18] is preferred. The calculation formula of the ultimate shear capacity of the column is calculated for the new type of confined concrete column and compared with the test results.

The test shows that the failure mode of reinforced high-strength concrete constrained with the PVC pipe column is shear baroclinic failure, and the stirrup bends when it breaks, and its strength is fully exerted. According to the current concrete standard structural column of China, the calculation formula of the ultimate shear capacity *V* is as follows:

(3) $$V=\frac{1.75}{\lambda +1.0}f_{t}bh_{0}+f_{yv}\frac{A_{sv}}{s}h_{0}+0.07N$$

where *λ* is the shear span ratio, *f*_t_ is the tensile strength of the concrete, *b* is the section width, *h*_0_ is the effective height of the section, *f*_yv_ is the yield strength of the stirrup, *A*_sv_ is the cross-sectional area of the stirrup, *s* is the pitch of the stirrup, and *N* is the axial pressure.

Equation (3) is used to calculate the specimen. The calculated result *V*_c_ was compared with the measured value *V*_t_, as shown in Table 6. As can be seen from the table, the measured values are larger than the calculated values, and the average ratio of *V*_t_ to *V*_c_ is 1.26. Therefore, it is feasible and safer to use the current standard calculation method to calculate the shear bearing capacity of the new type of confined concrete structure column. Owing to the lack of experimental data and the complexity of shear problems, the refined calculation method for the ultimate shear bearing capacity of this new type of confined concrete column needs to be further studied.

### 5.2. Superposition Method to Calculate Ultimate Shear Bearing Capacity

**Figure 14:**
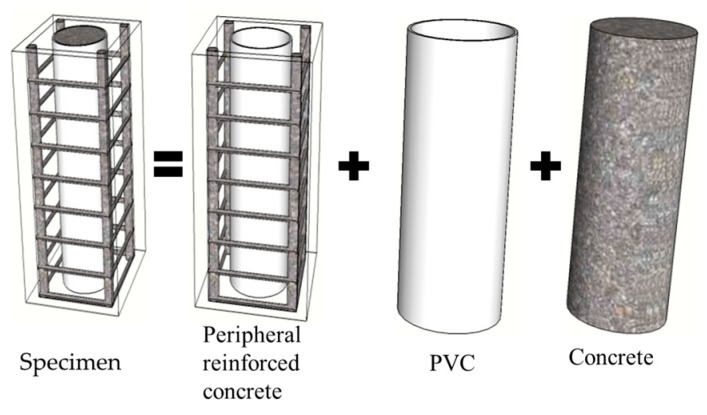
Force simplification model.

The shearing problem of reinforced concrete specimens is very complex. When it comes to the embedded PVC pipes, the calculation of the shear bearing capacity of reinforced high-strength concrete columns embedded PVC pipes is more complex. On the basis of the research results of the concrete-filled steel tube and composite structure at home and abroad [19,20,21], this paper ignores the bonding between the PVC pipe and high-strength concrete, and regards the shear capacity of high-strength concrete columns embedded PVC pipe as peripheral reinforced high-strength concrete and PVC pipe high-strength concrete, which can be divided into two parts: empty PVC pipe and high-strength concrete core in pipe., as shown in Figure 14. The formula of the shear capacity of the member is as follows:

(4) $$V=V_{T}+V_{rhc}$$

where *V* is the ultimate bearing capacity of reinforced high-strength concrete columns embedded pipes; *V*_T_ is the shear force of the PVC pipes (or steel tube) and high-strength concrete, and *V*_T_ = *V*_T1_ + *V*_T2_ + *V*_T3_, where *V*_T1_, *V*_T2_, and *V*_T3_ are the shear capacity of the pipe, the shear capacity of the concrete, and the axial pressure for the shear capacity of the component, respectively; and *V*_rhc_ is the shear force of periphery of the high-strength concrete.

(1) Shear Capacity of PVC Pipe High-Strength Concrete

At present, the systemic research of the calculation method for the shear capacity of PVC high-strength concrete is lacking. Considering that the mechanism of PVC pipe confining core concrete is similar to that of steel pipe, the shear strength of the PVC high-strength concrete force calculation model and method is established, which is based on the theory of shear performance in the concrete confined steel tube. In order to simplify the calculation, the following basic assumptions are made with reference to the research on the concrete-filled steel tube:

- Neglect the contribution of the PVC pipe hoop on the shear strength of concrete;
- The PVC pipe meets the von Mises yield criterion;
- Axial pressure is evenly distributed along the cross-section of the specimens.

It is assumed that the PVC pipe and the steel pipe satisfy the von Mises yield criterion, and the shear strength *f*_Ty_ ≈ 0.6*f*_T_, where *f*_T_ is the ultimate strength of the PVC pipe or the yield strength of the steel tube. When the shear span ratio *λ* = 0, the pipe is in a pure shear state; when the shear span ratio *λ* is more than 0.5, the pipe damage has typical bending failure characteristics. When the pipe is in these two specific critical states, the calculation formula of the shear bearing capacity *V*_T1_ can be obtained according to the classical mechanics [12].

(5) $$V_{T1}=\left\{ \begin{array}{l} \frac{f_{Ty}A_{y}}{2}=0.3f_{T}A_{T}\left(\lambda =0\right)\\ \frac{1+{\left(r_{1}/r_{2}\right)}^{2}}{8\lambda }f_{T}A_{T}\left(\lambda \geq 0.5\right) \end{array}\right.$$

where *V*_T1_ means the shear capacity, which includes *V*_P1_ as the shear capacity of the PVC pipe and *V*_a1_ as that of the steel tube; *A*_T_ is the cross-sectional area of the PVC pipe or the cross-sectional area of the steel tube; *r*_1_, *r*_2_ are the inner and outer diameters of pipe, respectively; and *λ* = *H*/*r*_2_, where H is the height of the pipe. When the shear span ratio is 0 < *λ* < 0.5, the shear capacity of the pipe is determined by interpolation.

**Figure 15:**
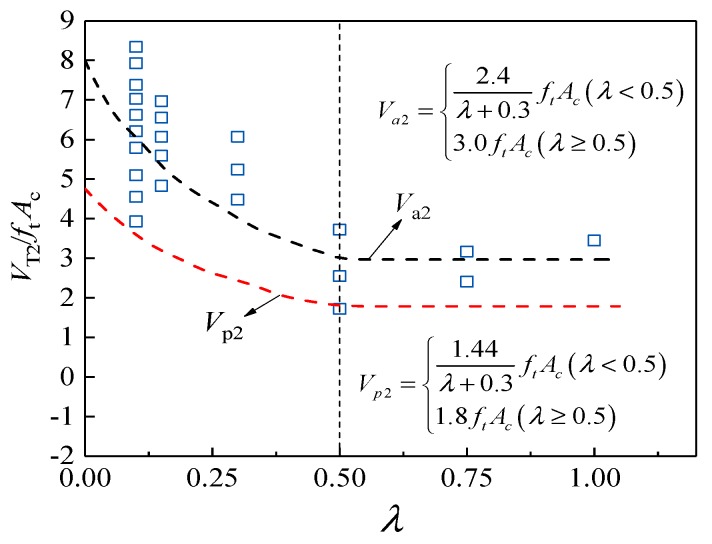
*V*_a2_ trend along with the shear span ratio.

The tests have shown that, when the concrete-filled steel tube reached the ultimate shear bearing capacity, the hoop strain value of the steel pipe was not large, which means that the restraining effect of the steel pipe on the core concrete was not very remarkable. Therefore, when calculating the shear strength of the core concrete, the beneficial effect of the steel tube on the improvement of the shear strength of the core concrete can be neglected. The measured shear bearing capacity of the steel core concrete *V*_a2_ trend along with the shear span ratio is shown in Figure 15. The curve can be obtained through the software [12].

(6) $$V_{a2}=\left\{ \begin{array}{l} \frac{2.4}{\lambda +0.3}f_{t}A_{c}\left(\lambda <0.5\right)\\ 3.0f_{t}A_{c}\left(\lambda \geq 0.5\right) \end{array}\right.$$

As the strength of the PVC pipe is low, it is recommended to take the lower limit envelope of the shear strength of the PVC core core with the shear span ratio curve lack of research data. The fitting expression of PVC shear bearing *V*_p2_ is as follows:

(7) $$V_{p2}=\left\{ \begin{array}{l} \frac{1.44}{\lambda +0.3}f_{t}A_{c}\left(\lambda <0.5\right)\\ 1.8f_{t}A_{c}\left(\lambda \geq 0.5\right) \end{array}\right.$$

In the same condition, when the axial pressure effect is still present in the tubular concrete column, the shear bearing capacity of the tubular concrete column will also increase. The added value is the contribution of the axial pressure. The contribution value of the axial pressure against shear is as follows:

(8) $$V_{T3}=\zeta N$$

where *N* is the axial pressure of the concrete pipe of the PVC pipe (steel tube). When *N* > 0.4 (*f*_T_*A* + *f*_c_*A*_c_), take *N* = 0.4 (*f*_T_*A* + *f*_c_*A*_c_) to calculate the PVC pipe (steel tube) shear capacity of concrete columns. *ξ* is the axial pressure influence coefficient of the shear capacity of pipe concrete.

The distribution of axial pressure in the confined high-strength concrete columns embedded PVC pipe (steel pipe) is very complicated. To simplify the calculation, it is assumed that the axial pressure is evenly distributed along the entire section of the specimen, that is, the axial pressure of the high-strength concrete part of the PVC pipe (steel tube) is *Ψ*_T_*N*, where *Ψ*_T_ is the area ratio of the high-strength concrete of the pipe, which refers to the ratio of the cross-sectional area *A*_Tc_ of the high-strength concrete of the pipe to the total cross-sectional area *A*.

**Figure 16:**
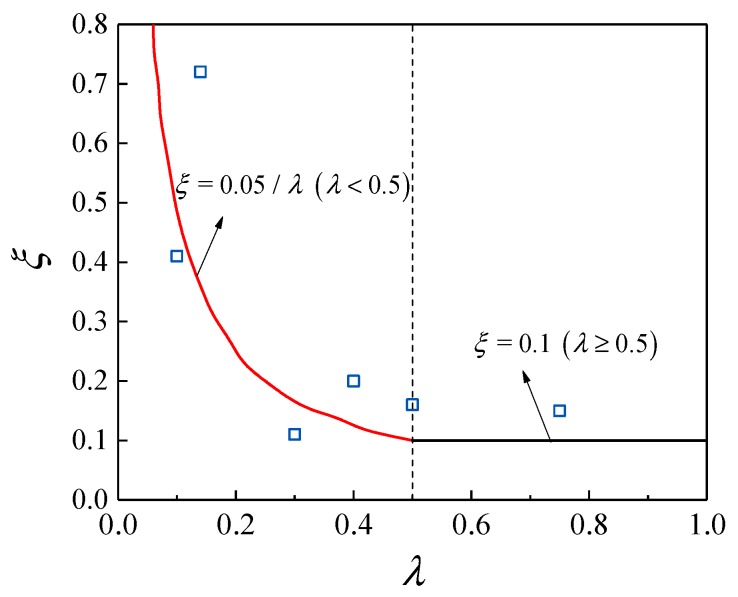
Axis pressure influence coefficient change curve.

Generally, the axial pressure influence coefficient *ξ* of the pipe concrete shear capacity decreases with the increase of the shear span ratio *λ* and the axial pressure ratio *n*, and the factors affecting *ξ* are more complicated. Figure 16 shows the curve of the axial pressure influence coefficient ξ, which is the shear capacity of the concrete filled steel tube. The expression is as follows [12]:

(9) $$\zeta =\left\{ \begin{array}{l} 0.05/\lambda \left(\lambda <0.5\right)\\ 0.1\left(\lambda \geq 0.5\right) \end{array}\right.$$

It can be seen from Figure 16 that, when the shear span ratio λ > 0.5, the change of the shear capacity of the high-strength concrete column of the steel tube is gradually stabilized, and the specific values are shown in Table 7. Considering the limitation of the ratio of enhanced shear bearing capacity to the total shear capacity with the increase in axial pressure and the view of simplifying the calculation and conservation, it is recommended that the concrete *ξ* of the steel tube (PVC pipe) is taken as 0.10.

(2) Shear Capacity of High-Strength Concrete

For the reinforced high-strength concrete columns embedded PVC pipe, in addition to the influence of axial pressure, the shear bearing capacity of the external reinforced high-strength concrete mainly includes the shear bearing capacity of high-strength concrete and the shear bearing capacity of the stirrup. Therefore, this part adopts Equation (10) for calculation:

(10) $$V_{rhc}=\frac{1.05}{\lambda +1.0}f_{t}\left(bh_{0}-A_{Tc}\right)+f_{yv}\frac{A_{sv}}{s}h_{0}+0.056\left(1-\psi_{T}\right)N$$

where *V*_rhc_ is the shear ultimate bearing capacity of the high-strength concrete part of the peripheral reinforcement; *f*_yv_ is the yield strength of the stirrup; *Ψ*_T_ is the area ratio of the high-strength concrete of PVC pipe and steel tube; and *A*_Tc_ is the cross-sectional area of the pipe concrete.

(3) Analysis of Calculation Results

According to the superposition method, the shear ultimate bearing capacity of reinforced high-strength concrete embedded with the PVC pipe is calculated as shown in Table 8. It can be seen from Table 8 that the mean value of the shear ultimate bearing capacity of reinforced high-strength concrete embedded with PVC pipe is 1.18 and the coefficient of variation is 7.4%. It can be seen that the calculation is safer. The calculation formula of the shear ultimate bearing capacity proposed by the above calculation model can be used to predict the ultimate shear bearing capacity of PVC pipes with low strength in the high-strength concrete short columns.

## 6. Conclusions

In this paper, the hysteretic behavior of embedded PVC pipe confined reinforced high strength concrete columns was investigated. The following conclusions can be drawn:

(1) When the shear span ratio is not large (*λ* ≤ 2.5), the failure mode of the reinforced high-strength concrete column embedded PVC pipe is shear barocliny failure. The influence of the axial compression ratio, shear span ratio, and other parameters on the failure mode was not obvious.
(2) Compared with the reinforced high strength concrete column restrained with the ordinary stirrup, the hysteretic curve of the high strength concrete column with the PVC pipe and steel tube inside is fuller, the peak load is increased, the strength attenuation in the later stage is slow, the decline section of the skeleton curve is gentle, the limit deformation is large, and the ductility is good.
(3) The restraint effect of the PVC pipe and steel tube is similar, which can provide an effective restraint effect and effectively enhance the seismic energy dissipation and deformation capacity of the reinforced high strength concrete column.
(4) For specimens with the same configuration of the PVC pipe, with the increase in the axial compression ratio and shear span ratio, the ductility becomes worse, and the value of PVC pipe diameter has an impact on its seismic performance. It is not that the larger the pipe diameter, the better; there is a problem with the value of the reasonable pipe diameter ratio.
(5) The ductility of the high-strength concrete column can be effectively improved by adopting measures of the internal PVC pipe, and the displacement ductility coefficient is greater than 3 in most cases. The lateral displacement angle of the limit exceeds the limit of the displacement angle between the layers of the concrete frame in the code.
(6) In order to facilitate the calculation of shear strength of the concrete column confined by the PVC pipe, a simplified calculation method is adopted. According to the formula of the current concrete specification, the safety surplus is about 25%.

## Figures and Tables

**Table 1:** Parameters of specimens.

Specimen	*L* (mm)	*b* × *h* (mm)	*λ*	*n*	Enhancements	Diameter	Longitudinal Reinforcement	Stirrup Form	Design Value of Concrete Strength
PVC-RHC-1	300	200 × 200	1.50	0.25	PVC Pipe	Φ110 × 3	4C18	B6@60	C75
PVC-RHC-2	300	200 × 200	1.50	0.45	PVC Pipe	Φ110 × 3	4C18	B6@60	C80
PVC-RHC-3	300	200 × 200	1.50	0.25	PVC Pipe	Φ75 × 2.3	4C18	B6@60	C70
CST-RHC	300	200 × 200	1.50	0.25	Round Steel Tube	Φ110 × 3	4C18	B6@60	C70
RHC	300	200 × 200	1.50	0.25	Stirrup	Φ6	4C18	B6@60	C70

Note: *L* represents the height of test specimens; *h* and *b* are the sectional height and width of column cross section, respectively; *λ* is the shear span ratio; *n* is the axial compression ratio. PVC, polyvinyl chloride; CST, circle steel tube; RHC, reinforced high-strength concrete.

**Table 2:** Concrete proportions.

Design of Value Concrete Strength	Material Content							
	Cement (kg/m^3^)	Sand (kg/m^3^)	Gravel (kg/m^3^)	Water (kg/m^3^)	Slag Powder (% of Cement Mass)	Silica Fume (% of Cement Mass)	Water Reducer (% of Cement Mass)	*f*_cu_ (MPa)
C75	482	664	1097	160	15	10	1.5	75.01
C80	533	745	1117	160	15	10	1.5	79.44

**Table 3:** Physical properties of steel bars and tubes.

Category	Specification	*f*_y_ (MPa)	*f*_u_ (MPa)	*E*_s_ (MPa)
Steel Bars	B6	277	380	1.798 × 10^5^
	C18	465	644	2.017 × 10^5^
Round Steel Tube	Φ110 × 3	392	499	2.191 × 10^5^
PVC pipe	Φ110 × 3	−	19.56	3315
	Φ75 × 2.3	−	20.20	3075

**Table 4:** Mechanical characteristics of loads and displacements.

NO.	Direction	Crack Point		Yield Point		Peak Point		Ultimate Point	
		*P*_cr_ /kN	Δ_cr_ /mm	*P*_y_ /kN	Δ_y_ /mm	*P*_m_ /kN	Δ_m_ /mm	*P*_u_ /kN	Δ_u_ /mm
PVC-RHC-1	Push	100	1.73	137.31	3.49	196.54	11.47	167.10	13.55
	Pull	80	2.30	110.70	4.51	145.54	9.55	123.70	12.66
	Average	90	2.02	124.01	4.00	171.04	10.51	145.4	13.11
PVC-RHC-2	Push	140	1.84	165.40	2.79	240.97	6.49	204.82	9.53
	Pull	140	2.69	149.36	2.92	187.25	6.00	159.16	9.18
	Average	140	2.27	157.38	2.86	214.11	6.25	181.99	9.36
PVC-RHC-3	Push	100	2.31	140.12	4.37	193.66	7.70	164.61	18.97
	Pull	100	3.23	108.90	4.17	139.77	9.00	118.81	20.35
	Average	100	2.77	124.51	4.27	166.72	8.35	141.71	19.66
CST-RHC	Push	120	2.38	134.27	2.98	201.21	9.91	171.03	17.31
	Pull	100	3.00	119.15	4.23	160.27	12.01	136.23	20.31
	Average	110	2.69	126.71	3.61	180.74	10.96	153.63	18.81
RHC	Push	100	3.95	136.72	6.16	182.00	9.96	179.66	12.30
	Pull	100	3.86	99.29	5.32	138.85	9.99	138.85	9.99
	Average	100	3.91	118.01	5.74	160.43	9.98	159.26	11.15

**Table 5:** Inter-story drift ratio and ductility of each characteristic point.

NO.	Direction	Crack Point	Yield Point	Peak Point	Ultimate Point	Ductility Factor
		*θ*_cr_/%	*θ*_y_/%	*θ*_m_/%	*θ*_u_/%	*μ*
**PVC-RHC-1**	Push	0.58	1.15	3.85	4.54	3.88
	Pull	0.77	1.49	3.23	4.17	2.81
	Average	0.68	1.32	3.54	4.36	3.35
PVC-RHC-2	Push	0.61	0.93	2.17	3.23	3.42
	Pull	0.89	0.97	1.59	3.03	3.14
	Average	0.75	0.95	1.88	3.13	3.28
PVC-RHC-3	Push	0.77	1.45	2.56	6.25	4.34
	Pull	1.08	1.39	3.03	6.67	4.88
	Average	0.92	1.42	2.80	6.46	4.61
CST-RHC	Push	0.79	0.99	3.33	5.88	5.81
	Pull	1.00	1.41	4.00	6.67	4.8
	Average	0.90	1.20	3.67	6.27	5.31
RHC	Push	1.32	2.04	3.33	4.17	2.00
	Pull	1.28	1.79	3.33	3.33	1.88
	Average	1.30	1.91	3.33	3.75	1.94

**Table 6:** Comparison of ultimate shear bearing capacity between the test value and calculated value.

NO.	*λ*	*b* (mm)	*h* (mm)	Stirrup Form	*f*_tk_ (MPa)	*f*_yv_ (MPa)	*N* (kN)	*V*_t_ (kN)	*V*_c_ (kN)	*V*_t_ /*V*_c_	Note
PVC-RHC-1	1.5	200	200	Φ6@60	4.62	277	480	196.54	163.43	1.20	*μ* = 1.26 *D* = 0.098 C.*V* = 7.8%
PVC-RHC-2	1.5	200	200	Φ6@60	4.80	277	900	240.97	167.99	1.43	
PVC-RHC-3	1.5	200	200	Φ6@60	4.62	277	480	193.66	163.42	1.19	
CST-RHC	1.5	200	200	Φ6@60	4.62	277	480	201.21	163.42	1.23	

**Table 7:** Axis pressure influence factor value.

*λ*	0.1	0.14	0.3	0.4	0.5	0.75
*ξ*	0.41	0.72	0.11	0.20	0.16	0.15

**Table 8:** Comparison of ultimate shear bearing capacity between the test value and calculated value.

NO.	*f*_tk_ (MPa)	*f*_yv_ (MPa)	*N* (kN)	*A*_Tc_ (mm^2^)	*V*_t_ (kN)	*V*_Tc_ (kN)	*V*_t_ /*V*_Tc_	Note
PVC-RHC-1	4.62	277	480	10,562.96	196.54	175.47	1.12	*μ* = 1.18 *D* = 0.087 C.*V* = 7.4%
PVC-RHC-2	4.80	277	900	10.562.96	240.97	180.53	1.33	
PVC-RHC-3	4.62	277	480	4973.89	193.66	144.02	1.13	
CST-RHC	4.62	277	480	10,562.96	201.21	177.14	1.14	
